# *Plasmodium falciparum* multiplicity of infection and pregnancy outcomes in Congolese women from southern Brazzaville, Republic of Congo

**DOI:** 10.1186/s12936-022-04105-w

**Published:** 2022-04-02

**Authors:** Jean Erick Massamba, Jean Claude Djontu, Christevy Jeannhey Vouvoungui, Charles Kobawila, Francine Ntoumi

**Affiliations:** 1 Fondation Congolaise Pour La Recherche Médicale (FCRM), Brazzaville, Republic of Congo; 2 Faculty of Sciences and Techniques, University Marien Ngouabi, Brazzaville, Republic of Congo; 3 Biotechnology Centre, University of Yaounde I, Yaoundé, Cameroon; 4 Institute of Tropical Medicine, University of Tübingen, Tübingen, Germany

**Keywords:** Malaria, *Msp-1*, Genetic diversity, Pregnancy, Republic of Congo

## Abstract

**Background:**

Investigating whether the multiplicity of *Plasmodium falciparum* infection (MOI) is related to pregnancy outcomes, is of interest in sub-Saharan area where malaria is highly endemic. The present study aimed to characterize the genetic diversity of *P. falciparum* in women at delivery from Southern Brazzaville, and investigate whether the MOI is associated with maternal anaemia, preterm delivery, or low birth weight.

**Methods:**

This was a cross sectional study carried out with samples collected between March 2014 and April 2015 from 371 women recruited at delivery at a Health Centre in southern Brazzaville, Republic of Congo. Matched peripheral, placental, and cord blood collected from each of the women at delivery were used for the detection of *P. falciparum* microscopic and submicroscopic parasitaemia, and parasite DNA genotyping by nested PCR.

**Results:**

From 371 recruited women, 27 were positive to microscopic malaria parasitaemia while 223 women harboured submicroscopic parasitaemia. All *msp*-*1* block 2 family allelic types (K1, MAD20 and RO33) were observed in all the three compartments of blood, with K1 being most abundant. K1 (with 12, 10, and 08 alleles in the peripheral, placental, and cord blood respectively) and MAD20 (with 10, 09, and 06 alleles in the respective blood compartments) were more diverse compared to RO33 (with 06, 06, and 05 alleles in the respective blood compartments). From the 250 women with microscopic and/or submicroscopic parasitaemia, 38.5%, 30.5%, and 18.4% of peripheral, placental and cord blood sample, respectively, harboured more than one parasite clone, and polyclonal infection was more prevalent in the peripheral blood of women with microscopic parasitaemia (54.5%) compared to those with submicroscopic parasitaemia (36.7%) (*p* = 0.02). The mean multiplicity of genotypes per microscopic and submicroscopic infection in peripheral blood was higher in anemic women (2.00 ± 0.23 and 1.66 ± 0.11, respectively) than in non-anaemic women (1.36 ± 0.15 and 1.45 ± 0.06, respectively) (*p* = 0.03 and 0.06). In logistic regression, women infected with four or more clones of the parasite were 9.4 times more likely to be anaemic than women harbouring one clone. This association, however, was only observed with the peripheral blood infection. No significant association was found between the MOI and low birth weight or preterm delivery.

**Conclusions:**

These results indicate that the genetic diversity of *P. falciparum* is high in pregnant women from southern Brazzaville in the Republic of Congo, and the multiplicity of the infection might represent a risk for maternal anaemia.

## Background

Pregnancy associated malaria (PAM) remains a major public health problem in sub-Saharan Africa, where approximately 50 million women become pregnant each year, and are at high risk of exposure to *Plasmodium falciparum*, the deadliest malaria parasite globally [1]. A significant decrease in the malaria-related morbidity and mortality has been reported since 2010, due to the intensification in preventive and control measures including the use of insecticide-treated bed nets (ITNs), indoor residual spray, the implementation of intermittent preventive treatment (IPT) especially in pregnant women as well as early diagnostic of cases, and generalized usage of artemisinin-based combination therapy (ACT) [1]. However, the rapid spread of insecticide and drug resistance fragilize efforts being undertaken [1], which would require to couple the current disease control tools with an effective vaccine for better impact. The development of an efficient vaccine against PAM requires an in-depth knowledge of the *Plasmodium* biology and host-parasite interactions.

One of the most important strategies used by *P. falciparum* to evade the immune response in pregnant women involves the sequestration of the parasite infected red blood cells in the placental tissue [2, 3]. The phenomenon is mediated by the adhesion of several infected erythrocyte variant surface antigens (VSA) to chondroitin sulfate A (CSA) expressed on the syncytiotrophoblast [4]. Although VAR2CSA is the best known and most studied *P. falciparum* infected erythrocytes variant surface protein, and the most promising target for vaccine development against PAM [3], there is an increased scientific evidence that non-VAR2CSA *Plasmodium* antigens mediated immune response also contribute to the malaria parasite clearance in pregnant women [5]. This suggests that the design of an effective vaccine against PAM should involve the combination of VAR2CSA-based antigen with others malaria vaccine candidates’ antigens, such as merozoite surface protein-1(MSP-1). Currently, the major obstacle for the development of such a multivalent vaccine is the extend of the genetic diversity of *P. falciparum* antigens, which limits the efficacy of acquired protective immunity against specific malaria parasite antigens [6]. In addition, previous studies associated the severity of malaria in pregnant women and non-pregnant individuals to others host-parasite factors, such as the multiplicity of infection (MOI) [7]. Merozoite surface protein genes have been used in several previous studies to characterize *P. falciparum* strains [8]. Among the *P. falciparum* blood-stage antigens, MSP-1 is the most expressed protein, and it also documented as a suitable markers to discriminate subpopulations of *P. falciparum* [9]. It is encoded by the *msp-1* gene located on chromosome 9, which contains 17 blocks of sequences flanked by conserved regions [9]. It is involved in erythrocyte invasion [10] and has also been identified as an important target for the development of anti-malaria vaccine [11]. Block 2, which is the most polymorphic part of *msp-1*, is grouped under three allelic families of K1, MAD20 and RO33 [12]. Fragment size polymorphism in the three block 2 allele families of *P. falciparum msp-1* has commonly been used as a molecular marker in studies of malaria transmission dynamics and host immunity in *P. falciparum* malaria [13–16].

In the areas of high malaria transmission, there is an increase of *Plasmodium* diversity and MOI in non-pregnant individuals [16, 17]. In addition, some studies reported an association between PAM and highly polymorphic parasites and multiclonal parasites [18, 19], while others showed the reverse [20, 21]. However, only few studies have been carried out on the genetic diversity of *P. falciparum* in Congolese pregnant women [22], and none of these reports has investigated their relationship with poor pregnancy outcomes, such as maternal anaemia, low birth weight, and preterm delivery. To complete current data on *P. falciparum* genetic diversity, this study aimed to characterize the genetic diversity of *P. falciparum* by investigating *msp1* block 2 polymorphisms, and determine whether the multiplicity of the infection is associated with poor pregnancy outcomes in women from Southern Brazzaville in the Republic of Congo.

## Methods

### Study site

This descriptive cross-sectional study was carried out from March 2014 to April 2015 at the Madibou integrated health centre, situated in the southern part of Brazzaville, in the Republic of Congo. The capital, Brazzaville, is located along the Congo River, in the south of the country. Since the country is located on the Equator, the climate is consistently humid year-round, with the average day temperature of 25 °C, and at night between 16 and 21 °C. The average yearly rainfall ranges from 1100 mm in the south to over 2000 mm in the central and north parts of the country. The rainy season which lasts 9 months, has two rainfall maxima: one in March–May and another in September–November. The malaria transmission in Brazzaville is perennial with *P. falciparum* as the predominant malaria parasite and *Anopheles gambiae* as the main mosquito vector.

### Study population and sample collection

A total of 371women aged 16 to 39 years old with no history of clinical malaria in the last two weeks before the delivery, and no history of fever, 48 h before the delivery or at the time of sample collection, were randomly recruited. Information on the mother’s health, estimated length of pregnancy, parity, age, baby birth weight, usage of anti-malarial drugs, IPTp-SP usage, and bed nets usage, were recorded using a standard questionnaire. The poor pregnancy outcomes investigated included preterm delivery (gestational age <37 weeks), low birth weight (birth weight <2.5 kg) and maternal anaemia (haemoglobin level ˂11 g/dL). Peripheral, placental and cord blood samples were collected in EDTA tubes from women immediately following delivery as previously described [23, 24]. A portion of the blood was used to prepare thick and thin film smears for microscopy and to measure haemoglobin levels. The remainder was stored at − 80 ºC until used for the parasite DNA genotyping by nested PCR.

### Diagnosis of malaria and determination of haemoglobin levels

Thick and thin blood smears were prepared for each peripheral and cord blood sample using Giemsa-Wright stain and read by two skilled microscopists to determine the presence or not of malaria parasites and the parasitaemia. Haemoglobin levels in maternal peripheral blood were determined using a haematologic analyzer.

### Genomic DNA extraction and genotyping

Parasite genomic DNA was extracted from collected blood samples using the QIAmp DNA mini kit according to manufacturer’s instructions (Qiagen GmbH, Hilden, Germany). Briefly, 200 µL of the whole blood was used to yield a nucleic acid elution volume of 60 µL and kept at − 20 °C until genotyping. Nested PCR genotyping was performed by amplification of the highly polymorphic Block 2 region of *msp-1*, considered to be the most informative genetic marker for the assessment of multiplicity of *P. falciparum* infection [25]. An initial amplification of the outer region of the gene was followed by individual nested PCR reactions using family specific primers for K1, MAD20, RO33. All reactions were carried out in a final volume of 20 μl containing 1X of MgCl2 free buffer, 2 mM of MgCl2, 125 μM dNTPs, 250 nM of each primer (Table 1) and 0.4 U of Taq polymerase. In the first round reaction, 1 μl of genomic DNA was added as a template and the amplification performed in a thermocycler (Master Cycler X50 a Eppendorf AG, Hamburg, Germany) using: initial denaturation at 95 °C for 3 min, followed immediately by 25 cycles of denaturation at 94 °C for 45 s, annealing at 55 °C for 1 min and extension at 72 °C for 2 min. The final cycle had a prolonged extension at 72 °C for 5 min. In the nested reaction, 0.5 μl of the primary PCR product was added as DNA template and the amplification performed similarly to the first round except that the primer concentrations were doubled, the annealing done at 61 °C for 2 min and the cycles of denaturation-annealing-extension increased to 30. The positive controls (*P. falciparum* laboratory strain 3D7) and DNA-free negative controls were included in each series of reactions. Five microlitres of each of the PCR products were loaded on to a 1.5% agarose gel, stained with Syber Green, separated by electrophoresis and visualized under UV light (BIORAD Gel doc TM 201 EZ Imager, USA), and fragment sizes estimated by comparison to DNA ladder. The prevalence of each allelic type was determined as the presence of PCR products for the type in the total number of amplified bands. The MOI, the number of genotypes per infection was estimated as the average number of PCR fragments per individual, by dividing the total number of *msp-1* fragments detected by the number of positive samples as described previously [26]. Monoclonal infection was defined as the presence of only one allele of the three major *msp-1* types in the sample while isolates with two or more genotype were considered as polyclonal infection [27].

**Table 1:** Primers for PCR genotyping of block 2 region of msp-1

Amplication allele primer		Primer sequence
Primary PCR	NA	Forward 5’-CTAGAAGCTTTAGAAGATGCAGTATTG-3’ Reverse 3’-CTTAAATAGTATTCTAATTCAAGTGGATCA-5’
Secondary PCR	K1	Forward 5’-AAATGAAGAAGAAATTACTACAAAAGGTGC-3’ Reverse 3’-GCTTGCATCAGCTGGAGGGCTTGCACCAGA-5’
	MAD20	Forward 5’-AAATGAAGGAACAAGTGGAACAGCTGTTAC-3’ Reverse 3’-ATCTGAAGGATTTGTACGTCTTGAATTACC-5’
	RO33	Forward 5’-TAAAGGATGGAGCAAATACTCAAGTTGTTG-3’ Reverse 3’-CATCTGAAGGATTTGCAGCACCTGGAGATC-5’

### Statistical analysis

Graph Pad Prism (version 6.0.1) and XLSTAT (version 2011.2.08) software were used for the statistical analyses. Continuous variables (age, gravidity, haemoglobin level, gestational age, birth weight, IPT-doses) are reported as means ± standard deviations (SD) or medians with interquartile range (IQR). Differences between groups were compared using unpaired Student’s t test for normally distributed continuous data while Mann–Whitney Rank Sum test or Kruskal–Wallis test was used for non-normal distributed continuous data. Categorical variables (malaria infection status, preterm term delivery, low birth weight, IPT-SP and ITN usage) were reported as percentages and were compared using Fisher’s exact test or Chi Square test. Logistic regression analyses were performed where preterm delivery, low birth weight, maternal anaemia were entered in the model as dependent variables and the infection with 1clone, 2–3 clones, and ≥ 4 clones as independent variables. The polyclonal infection status (i.e. infection with more than 1 clone of the parasite in the peripheral and/or placental blood) was also entered in the models as dependent variable when mother age, gravidity, using bed net and IPT-SP were entered as independent variables. Two-sided p values ˂ 0.05 were considered statistically significant.

## Results

### Characteristic of the study population

The characteristics of the study participants are summarized on Table 2. Overall, 371women at the delivery with no malaria symptoms were enrolled, mainly with submicroscopic (223, 60.1%) and microscopic parasitaemia (27, 7.3%), providing a total of 250 infected women used for the analyses of *P. falciparum* genetic diversity. No case of microscopic infection was detected in the cord blood, and 22 (81.5%) women had microscopic parasitaemia in both peripheral and placental blood. Of 223 women with submicroscopic parasitaemia, 68 (30.5%) women were positive for both peripheral and placental blood while only 27 (12.1%) women were positive for all the three blood compartments. Women with microscopic malaria parasitaemia (median age, 23 years) were younger than those with submicroscopic parasitaemia (median age, 25 years) (*p* = 0.027). The mean parity (2.1) and gravidity (2.2) were lower (*p* = 0.05 and 0.008 respectively) in women with microscopic parasitaemia than in submicroscopic parasitaemia positive women (2.7 and 3.2 respectively). Maternal median haemoglobin level was lower in women with microscopic parasitaemia (10.05 g/dL) than in submicroscopic parasitaemia positive group (11.60 g/dL) (*p* = 0.011), and the percentage of anaemia was higher in the group of women harbouring microscopic parasitaemia (*p* = 0.02). The mean birth weight as well as the frequency of preterm delivery were not significantly different between women with microscopic (3081 g and 7/27, 29.5%, respectively) and submicroscopic parasitaemia (3130 g and 39/223, 17.5%), respectively (*p* = 0.62 and 0.28, respectively). The mean number of IPTp-SP doses taken by submicroscopic parasitaemia positive women was higher than that of microscopic parasitaemia positive women (*p* = 0.01), but the proportion of women using bed nets was greater in microscopic parasitaemia positive women compared to submicroscopic parasitaemia group (*p* < 0.0001).

**Table 2:** General characteristics of the study population

Variables	Microscopic infected women n = 27	Submicroscopic infected women n = 223	*p* value
Age in years (median with IQR)	23 (20; 27)	25 (21; 31)	0.027
≤ 20 years	7/27 (25.9%)	44/223 (19.7%)	0.45
21–30 years	18/27 (66.7%)	118/223 (52.9%)	0.22
> 30 years	2/27 (7.4%)	60/223 (26.9%)	0.03
Parity (mean ± SD)	2.1 ± 1.0	2.7 ± 1.5	0.05
Gravidity (mean ± SD)	2.2 ± 1.1	3.2 ± 1.7	0.008
Primigravidae	8/27 (29.6%)	42/223 (18.8%)	0.20
Secundigravidae	8/27 (29.6%)	47/223 (21.1%)	0.33
Multigravidae	11/27 (40.7%)	134/223 (60.0%)	0.06
Haemoglobin level in (g/dL) (median with IQR)	10.05 (8.45; 12.60)	11.60 (10.30; 13.00)	0.011
Maternal anaemia (Hb < 11 g/dL)	16/26 (61.5%)	80/221 (36.2%)	0.02
Gestational age in weeks (median with IQR)	38 (36; 39)	38 (37; 39)	0.06
msp-1 DNA positive individuals	27 (100%)	223 (100%)	1
Preterm delivery (GA < 37 weeks)	7/27 (25.9%)	39/223 (17.5%)	0.28
Birth weight in g (mean ± SD)	3081 ± 0.449	3130 ± 0.482	0.62
Baby low birth weight	2/27 (7.4%)	17/223(7.6%)	1.00
IPT-SP usage			
Doses of SP (mean ± SD)	1.2 ± 0.9	1.7 ± 1.0	0.020
0 Dose of SP	6/27 (22.2%)	54/343 (15.7%)	0.41
1 Dose of SP	13/27 (48.1%)	90/343 (26.2%)	0.02
2 Doses of SP	4/27 (14.8%)	91/343 (26.5%)	0.25
3 Doses of SP	4/27 (14.8%)	108/343 (31.9%)	0.08
Women using ITNs	27/27 (100%)	222/343 (64.7%)	< 0.0001

*IQR* interquartile ranges), *SP* Sulfadoxine-pyrimethamine, *ITNs* insecticide-treated net

### Distribution of *P. falciparum msp-1* block 2 allelic diversity and haplotype frequency in women at delivery

**Fig. 1:**
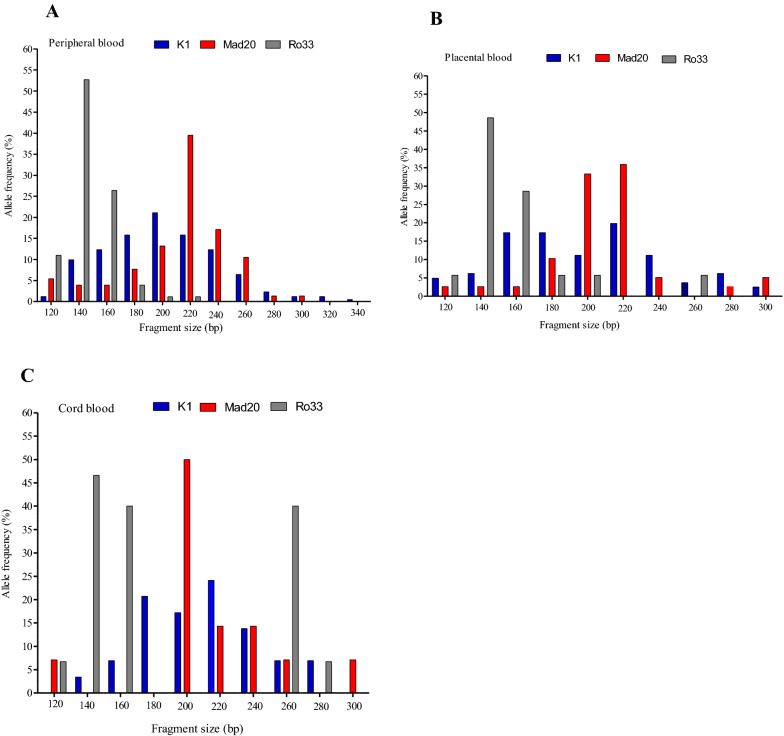
Distribution of fragment size polymorphisms and allele frequency of *msp1* block 2 allele families. **A** peripheral blood, **B** placental blood, **C** Cord blood

Length polymorphism was assessed in 250 *P. falciparum* field isolates. The number of genotypes observed at each blood compartment is shown in Fig. 1. All *msp1* family specific allelic types i.e. K1, MAD 20 and RO33 were observed in the different blood compartment. The gene was more diverse in the peripheral blood where twenty eight (28) different alleles were observed, representing K1 (12 alleles), MAD20 (10 alleles) and RO33 (6 allele), than in the placental blood with 25 different alleles, representing K1 (10 alleles), MAD20 (9 alleles), R033 (6 alleles), and in cord blood with 19 alleles, K1 (8 alleles), MAD20 (6 alleles), R033 (5 alleles). In the peripheral blood compartment, 171 (51%) individuals harboured K1 type allele compared to 76 (22%) for MAD20 and 91 (27%) for RO33 type allele. The similar trend was observed in the placental blood compartment where 70 (49%) individuals harboured K1 type allele compared to 37 individuals (26%) for MAD20, and 35 (25%) individuals for RO33 type allele; and in cord blood compartment where 29 (50%) individuals harboured K1 type allele compared to 14 individuals (24%) for MAD20, and 15 (26%) individuals for RO33 type allele.

### Variation in *msp-1* block 2 allelic family with the women gravidity

**Fig. 2:**
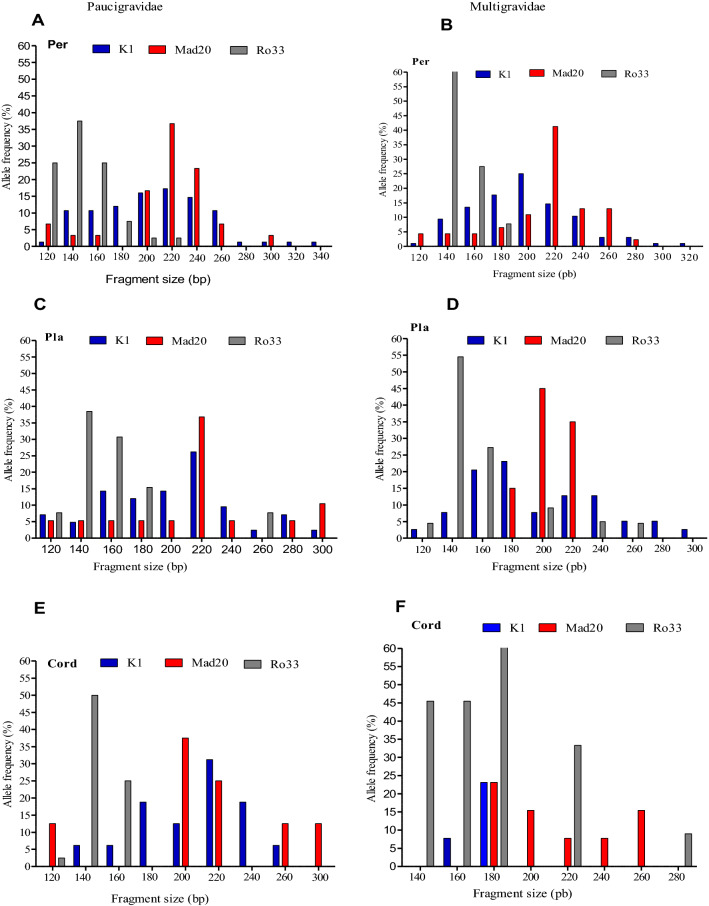
variation of fragment size polymorphisms and allele frequency of *msp1* block 2 allele families in peripheral (**A**, **B**), placental (**C**, **D**) and cord (**E**, **F**) blood between paucigravid and multigravid women at delivery with asymptomatic malaria infection

The frequency distribution of the *msp-1* bloc 2 family alleles in paucigravid (1or 2 gravities) and multigravid (more than two gravidities) women are presented on Fig. 2. In general, no significant difference on the diversity of these family alleles was observed between paucigravid and multigravid women: K1 (12 vs.11 alleles), MAD20 (8vs.9 alleles), and RO33 (6 vs.3 alleles) for the peripheral blood compartment; K1 (10 v.s10 alleles), MAD20 (3vs.8 alleles), and RO33 (5 vs.6 Alleles) for placental blood compartment, and K1 (7 vs.2 alleles), Mad20 (5vs. 5 alleles), and RO33 (3 vs. 5 Alleles) for cord blood. The frequencies of K1, MAD20, and RO33 type alleles in the peripheral blood compartment was 83% (75/90), 33% (30/90), and 44% (40/90) respectively among paucigravid women compared to 75% (96/128), 36% (46/128), and 40% (51/128) respectively for multigravid women. These frequencies were 84% (42/50), 38% (19/50), and 26% (13/50) respectively among paucigravid women compared to 67% (39/58), 34% (20/58), and 38% (22/58) respectively among multigravid women, for the placental blood compartment, and 70% (16/23), 35% (8/23), and 17% (4/23) respectively among primigravid women compared to 50% (13/26), 23% (6/26), and 42% (11/26) respectively among multigravid women, for the cord blood compartment.

### Variation in *msp-1* block 2 allelic family with the parasitaemia phenotype

**Fig. 3:**
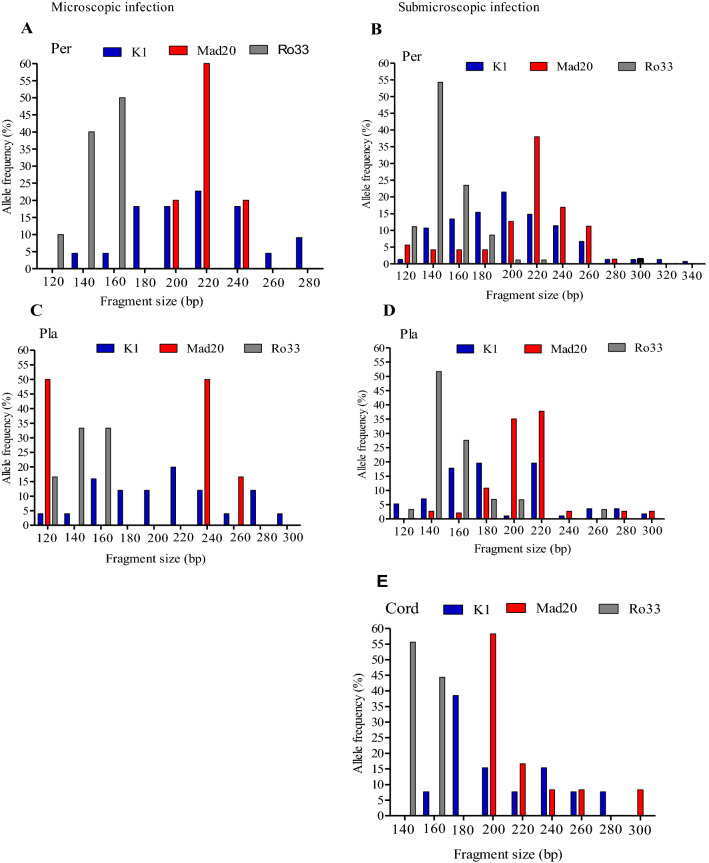
Variation of fragment size polymorphisms and allele frequency of *msp1* block 2 genes by infection phenotype status. **A**, **B** peripheral blood; **C**, **D** placental blood; **E** cord blood

The distribution and frequency of the msp1 bloc 2 family alleles in microscopic parasitaemia positive and submicroscopic parasitaemia positive women are presented on Fig. 3 and Table 3. In general, the *msp1* bloc 2 family alleles was less diverse in microscopic parasitaemia positive women compared to submicroscopic parasitaemia positive contreparts: K1 (8 vs. 12 alleles), MAD20 (3 vs. 10 alleles), and RO33 (3 vs. 6 alleles) for the peripheral blood compartment; K1 (10 v.s10 alleles), MAD20 (3 vs. 8 alleles), and RO33 (3 vs. 6 Alleles) for placental blood compartment (Fig. 3). However, the frequencies of K1, MAD20, and RO33 type alleles in the peripheral blood compartment were 100% (22/22), 23% (5/22), and 45% (10/22) respectively in microscopic parasitaemia positive women compared to 76% (149/196), 36% (71/196), and 41% (81/196) respectively among the submicroscopic parasitaemia positive women. These frequencies were 86% (19/22), 9% (2/22), and 27% (6/22) respectively among microscopic parasitaemia positive women compared to 60% (51/85), 41% (35/85), and 34% (29/85), respectively, in submicroscopic parasitaemia positive women, for the placental blood compartment, and 42% (13/31), 39% (12/31), and 29% (9/31), respectively, in submicroscopic parasitaemia positive women for the cord blood compartment. In general, the prevalence of polyclonal and monoclonal infections did not significantly differ trough the different blood compartments or between microscopic and submicroscopic groups (Table 3).

**Table 3:** Variation in *msp 1* block 2 diversity between microscopic and sub microscopic malaria infection

MSP1 block 2 allele type	Microscopic infection			Submicroscopic infection					
	Per n = 22	Pla n = 22	*p*-value	Per n = 196	Pla n = 85	Cord n = 31	*P*-value	a	b
K1	22 (100)	19 (86.4)	0.03	149 (76.0)	51 (60)	13 (41.9)	0.02	0.0004	< 0.0001
Mad 20	5 (23.0)	2 (9.1)	0.36	71 (36.2)	35 (41.2)	12 (38.7)	0.52	0.34	0.002
R033	10 (45.4)	6 (27.3)	0.05	81(41.3)	29 (34.1)	9 (29.0)	0.28	0.82	0.34
Monoclonal infection	10 (45.4)	15 (68.2)	0.19	124 (63.3)	60 (70.6)	27 (87.1)	0.02	0.11	0.62
Polyclonal infection	12 (54.5)	8 (36.4)	0.19	72 (36.7)	25 (29.4)	4 (12.9)	0.02	0.11	0.62
K1 + Mad 20	5 (22.7)	1 (4.6)	NA	36 (18.4)	14 (16.5)	2 (6.5)	0.25	0.57	0.60
K1 + R033	10 (45.4)	4 (18.2)	0.02	39 (19.9)	16 (18.8)	1 (3.2)	0.07	0.01	0.18
Mad20 + R033	3 (13.6)	0 (0)	NA	25 (12.8)	9 (10.6)	1 (3.2)	0.28	1.00	NA
K1 + Mad20 + R033	3 (13.6)	0 (0)	NA	17 (8.7)	6 (7.1)	0 (0)	0.22	0.43	NA

*Per* peripheral blood, *Pla* Placental blood, *Cord* Cord blood; ^a,b^significance of differences between microscopic and submicroscopic malaria infection in the proportion of K1, MAD20, RO33, polyclonal infection, and monoclonal infection from the peripheral, and placenta blood respectively; *NA* Non applicable

### MOI in relation with the socio-demographic status and poor pregnancy outcomes

For both microscopic and submicroscopic malaria infections, a majority of the infection was with a single *P. falciparum* genotype. However, an infection with all the 3 *msp1* bloc 2 family alleles was observed in 13.6% of peripheral blood samples harbouring microscopic parasitaemia, and in 8.7%, and 7.1% of peripheral and placental blood harbouring submicroscopic malaria parasitaemia (Table 3). The overall MOI ± SD of genotypes per infection was found to be 1.68 ± 0.72 (range: 1–3) in the peripheral blood, 1.43 ± 0.66 (range: 1–3) in the placental blood, and 1.33 ± 0.59 (range: 1–3) in the cord blood compartment for women with microscopic parasitaemia, and 1.55 ± 0.79 (range: 1–4) in the peripheral blood, 1.45 ± 0.74 (range: 1–4) in the placental blood, and 1.20 ± 0.46 (range: 1–3) in the cord blood compartment for women with submicroscopic parasitaemia. The MOI was similar (p = 0.72) across the different blood compartments and age groups. Moreover, there was not significant variation in MOI with low birth weight, preterm delivery, IPT-SP, and ITN usage. Nevertheless, the MOI was higher in anemic women with microscopic and submicroscopic parasitaemia (p = 0.03 and 0.06) than in non-anemic women counterparts (Table 4). In logistic regression, women infected with 4 or more clones of *P. falciparum* were 9.4 times more likely to be anaemic than women harbouring 1 clone. This association, however, was only observed with the peripheral blood infection. In this sub-group, the odds ratio (for anaemia in the group harbouring ≥ 4 clones compared with those with only one clone) was 9.4 (95% CI = 1.1–82.5), compared to 0.3 (95% CI = 0.3–8.9) for placental blood infection, and 1.8 (95% CI = 0.3–8.9) for cord blood infection. The odds ratio for preterm delivery and low birth weight did not significantly change with increased number of parasite clones per infection (Table 5). In addition, no significant association was found between the polyclonal infection (in the peripheral or/and placental blood) and mother age, gravidity, IPT-SP usage, or bed net usage (Table 6).

**Table 4:** Mean multiplicity of infection as the function infection phenotype

	Microscopic infection				Submicroscopic infection						*P*-value	
	n	Per	n	Pla	n	Per	n	Pla	n	Cord	a	b
Age group												
≤ 20	4	2.25 ± 0.50	5	1.80 ± 0.83	39	1.46 ± 0.85	16	1.25 ± 0.77	8	1.13 ± 0.35	0.07	0.18
21–30	16	1.44 ± 0.63	17	1.29 ± 0.58	104	1.56 ± 0.80	46	1.54 ± 0.78	18	1.11 ± 032	0.56	0.23
> 30	2	2.5 ± 0.50	1		53	1.50 ± 0.74	23	1.34 ± 0.71	5	1.12 ± 0.44	NA	NA
*P* value		NA		NA		0.80		0.34		0.88		
Gravidity												
1–2	12	1.75 ± 0.62	14	1.43 ± 0.64	78	1.59 ± 0.90	36	1.50 ± 0.81	15	1.17 ± 0.39	0.55	0.77
> 2	10	1.60 ± 0.84	9	1.44 ± 0.72	118	1.40 ± 0.71	49	1.39 ± 0.73	19	1.11 ± 0.31	0.62	0.83
*P* value		0.63		0.96		0.36		0.50		0.63		
SP												
0–1 dose	14	1.79 ± 0.21	15	1.40 ± 0.16	82	1.54 ± 0.09	41	1.5 ± 0.14	17	1.12 ± 0.08	0.29	0.66
2–3 doses	8	1.50 ± 0.18	8	1.50 ± 0.27	114	1.52 ± 0.07	44	1.36 ± 0.58	2	1.0 ± 0.0	0.95	0.58
*P* value		0.38		0.74		0.86		0.37				
ITN usage	15	1.67 ± 0.21	14	1.29 ± 0.16	125	1.48 ± 0.06	53	1.43 ± 0.10	23	1.17 ± 0.08	0.35	0.48
No ITN	7	1.71 ± 0.18	9	1.67 ± 0.24	71	1.60 ± 0.10	32	1.44 ± 0.15	8	1.0 ± 0.0	0.75	0.46
*P* value		0.89		0.18		0.29		0.98				
Anaemia	11	2.00 ± 0.23	12	1.42 ± 0.19	71	1.66 ± 0.11	29	1.38 ± 0.13	12	1.17 ± 0.11	0.27	0.87
No anaemia	11	1.36 ± 0.15	11	1.45 ± 0.21	125	1.45 ± 0.06	56	1.46 ± 0.11	19	1.10 ± 0.07	0.68	0.97
*P* value		0.03		0.89		0.06		0.63		0.63		
LBW	2	2.00 ± 1.00	1		14	1.57 ± 0.27	8	1.50 ± 0.38	2	1.00 ± 0.0	NA	NA
No LBW	20	1.65 ± 0.15	22	1.46 ± 0.14	182	1.52 ± 0.06	77	1.43 ± 0.08	29		0.48	0.88
*P* value		NA		NA		0.82		0.80		NA		
PTD	5	1.80 ± 0.44	6	1.17 ± 0.78	33	1.49 ± 0.15	18	1.22 ± 0.10	4	1.0 ± 0.0	0.44	0.78
No PTD	17	1.40 ± 0.19	17	1.53 ± 0.17	163	1.53 ± 0.06	67	1.49 ± 0.10	27	1.15 ± 0.09	0.48	0.86
*P* value		0.37		0.26		0.75		0.18		0.62		

NA: non-applicable; Per: peripheral blood, Pla: Placental blood, Cord: Cord blood; SP: Sulfadoxine-pyrimethamine, ITN: insecticide-treated net; LBW: low birth weight; PTD: preterm delivery; a, b: significance of differences in the multiplicity of the infection in peripheral, and placenta blood respectively between women with microscopic and submicroscopic malaria infection

**Table 5:** Odds of the *P. falciparum* number of clones per infection on the maternal anaemia, preterm delivery, low birth weight in women at delivery

Multiplicity of P. falciparum infection	n (%) PTD	Odds ratio (CI. 95%)	*P* values	n (%) LBW	Odds ratio (CI. 95%)	*P* values	n (%) Anaemia	Odds ratio (CI. 95%)	*P* values
Peripheral blood									
1 clone (n = 135)	25 (18.5)	Reference		10 (7.4)	Reference		47 ( 34.8)	Reference	
2–3 clones (n = 83)	12 (15.6)	0.7 (0.4–1.6)	0.44	4 (5.2)	0.6 (0.2–0.2.1)	0.75	36 (43.3)	1.4 (0.8–2.5)	0.25
≥ 4 clones (n = 6)	1 (16.6)	0.8 (0.1–7.9)	0.90	1 (16.6)	2.1 (0.2–19.0)	0.52	5 (83.3)	9.4 (1.1–82.5)	0.02
Placental blood									
1 clone (n = 58)	15 (25.9)	Reference		4 (6.9)	Reference		21 (36.2)	Reference	
2–3 clones (n = 31)	5 (16.1)	0.5 (0.2–1.7)	0.33	1 (3.2)	0.45 (0.1–4.2)	0.48	12 (38.7)	(0.5–2.7)	0.82
≥ 4 clones (n = 2)	0 (0)	0.6 (0.0–12.4)	0.71	1 (50)	13.5 (0.7–258)	0.08	0 (0)	0.35 (0.1–7.6)	0.50
Cord blood									
1 clone (42)	6 (14.3)	Reference		1(2.4)	Reference		18 (42.9)	Reference	
2–3 clones (7)	0 (0)	0.4 (0.0–7.4)	0.52	0 (0)	0.2 (0.0–7.2)	0.41	4 (57.1)	1.8 (0.3–8.9)	0.49

*PTD* preterm delivery, *LBW* low birth weight, *CI* confident interval

**Table 6:** Factors associated with *P. falciparum* polyclonal infection in woman at delivery

	n (%) polyclonale^a^ infection	Non adjusted odds ratio (CI. 95%)	Adjusted odds ratio (CI. 95%)	*p* values*
Mother age				
≤ 20 years (51)	16 (35.3)	Reference	Reference	
21–30 years (138)	63 (45.6)	1.63 (0.88–3.01)	1.65 (0.87–3.09)	0.119
>30 years (62)	25 (40.3)	1.24 (0.61–2.52)	1.25 (0.60–2.59)	0.550
Gravidity:				
1–2 (106)	47 (44.3)	Reference	Reference	
≥ 3 (145)	59 (40.7)	0.86 (0.52–1.43)	0.90 (0.53–1.53)	0.563
IPT-SP usage				
0–1 dose (117)	44 (37.6)	Reference	Reference	
2–3 doses (136)	58 (42.6)	1.04 (0.63–1.72)	1.02 (0.61–1.72)	0.925
ITNs usage:				
Yes (162)	67 (41.3)	Reference	Reference	
No (89)	39 (43.8)	0.90 (0.53–1.52)	0.89 (0.52–1.51)	0.659

*IPT-SP* intermittent preventive treatment using sulfadoxine-pyrimethamine, *CI* confident interval, *ITNs* insecticide-treated bed nets. *P values for adjusted Odds ratio

^a^In at least one of the peripheral, or placental blood compartment

## Discussion

Malaria in pregnancy remains a major health burden in sub-Saharan Africa, despite the use of sulfadoxine-pyrimethamine (SP) as the intermittent preventive treatment (IPT) strategy during pregnancy [1]. Patients with severe malaria are more likely to have multiclonal infection, and high multiplicity of *P. falciparum* infections might represent a major threat for the effectiveness of any malaria drug. However, data on the diversity of natural *Plasmodium* population in Congolese pregnant women under IPT-SP are limited. In addition, none of previous studies carried out in the Republic of Congo have investigated the relationship between the genetic diversity of the parasite and poor pregnancy outcomes such as maternal anaemia, low birth weight, and preterm delivery. The present study aimed to characterize the genetic diversity of *P. falciparum* by investigating *msp-1* block 2 polymorphisms and to evaluate the association between the MOI and pregnancy outcomes in women from Southern Brazzaville, Republic of Congo.

The results of this study indicate that *msp-1* block 2 family allelic types (K1, MAD20 and RO33) were observed in all the three compartments of blood, with K1 being most abundant followed by MAD20. Studies in other malaria endemic countries including neighbouring Gabon [28], Nigeria [29] as well as in Asia as far as Lao PDR [30] and India [31] have consistently shown that K1 type allele is more prevalent than MAD20 and RO33, in contrast to studies from Sudan and Malaysia where RO33 type allele was predominant [7, 27]. In addition, K1 and MAD20 type alleles in the present study were more diverse compared to RO33, and the infection with more than one parasite clone was common in all blood compartments. Studies have reported that there is genetic recombination during the sexual phase of the life cycle of the malaria parasite, and this randomly recombination of alleles enhances the diverse nature of the parasite [32, 33]. However, changing environments, drug pressure, and host immune responses might also represent potential causes of the genetic diversity of *P. falciparum* [34–36].

The highly diverse nature of *P. falciparum* observed in the current study is indicative of high transmission of malaria infection in the area and suggest a great potential for the development of resistance to SP which may render it less effective as a preventive treatment. Otherwise, although some authors believe that high biodiversity of malaria parasite might be benefit for the naturally acquired immunity to malaria since it gives the chance to subjects to be exposed to several different malaria antigens strains, the extension of the genetic diversity of *P. falciparum* antigens limits the efficacy of acquired protective immunity to a specific malaria candidate vaccine [6]. Thus, the high diversity of *P. falciparum* might also compromise the success of an anti-malarial vaccine implementation in this study area.

The *msp1* type genes were more diverse in peripheral blood compared to placental and cord blood. This data are different from those reported in Sudanese women, showing higher diversity of the gene in the placental blood compartment [37], but confirm the previous findings suggesting that the profile of *P. falciparum* strains circulating in the peripheral blood of pregnant women might differ from that found in the placental and cord blood. Previous studies from high malaria transmission settings have reported several strains of *P. falciparum* circulating in pregnant women among which some were more prevalent in the placental blood in comparison to peripheral blood compartment. The difference observed between the current study and the report [37] on the variation of *msp1* gene diversity between the different bloods compartments might be due to the difference in *P. falciparum* strains circulating in Sudan in comparison with the parasite strains in circulation in the Republic of Congo.

Conversely to some of previous reports that did not find any association between malaria parasite density and its genetic diversity [30, 38], the different *msp1* family alleles in this study were more diverse in submicroscopic parasitaemia positive women, but more frequent in microscopic parasitaemia positive women. This results are in line with previous findings suggesting that high diversity of *P. falciparum* increases the naturally acquired antibody repertory which might reduce the level of parasitaemia [6]. These findings from previous reports may also explain the high proportion of women using bed net found in this study in microscopic parasitaemia positive group compared to submicroscopic parasitaemia positive group, since bed net usage might be associated with limited exposure to the parasite antigens.

However, the higher frequency of the alleles observed in the microscopic parasitaemia positive women in comparison with women harbouring submicroscopic parasitaemia was unexpected, and may be explained by the high parasite density in the first group that eases its detection. Subsequent pregnancies in high malaria transmission settings are associated with high exposure to PAM which increases the naturally acquired antibody mediated immunity against PAM- specific *P. falciparum* strains. This corroborate the findings of this study showing lower risk of microscopic infection in older women, with high number of gravidity compared to younger women with low gravidity. Accurate detection of microscopic *P. falciparum* infection remains a major challenge in pregnant women who commonly harbour low parasitaemia, likely due to ITP-SP usage which reduces the parasite replication, and to ability of parasite to sequester in the placental tissue. These observations might also explain the high prevalence of submicroscopic parasitaemia (60.1%) found in the present study. Nevertheless, it is commonly known that the detection cut-off of malaria parasite by microscopic examination of Giemsa-stained thick blood film significantly varies with microscopists, and the rate of missed detection increases with low parasitaemia infection.

The frequency of polyclonal infection in the peripheral blood was higher in women with microscopic parasitaemia (54.5%) compared to those with submicroscopic parasitaemia (36.7%). These findings suggest that pregnant women having polyclonal *P. falciparum* infection are at high risk of suffering microscopic parasitaemia malaria infection. The analysis of the relationship between the biodiversity of *P. falciparum* in women and poor pregnancy outcomes, showed that maternal anaemia was more prevalent in women with high MOI. No significant association was found between the parasites MOI and preterm delivery or low birth weight. In addition, SP or insecticide treated bed nets (ITNs) usage as well as mother age and gravidity had no significant influence on the *msp1* genes diversity in the current study, in contrast to a previous study carried out in Sudan, which reported significant higher multiplicity of infection identified by at least two alleles of *msp-1* among paucigravidae compared to multigravidae [39]. The main limitation of this study included the non-use of placental tissue impression smear for the diagnosis of placental malaria which would have increased the prevalence of microscopic infection. Other limitations of the study include the low sample size, especially in different groups of women with anaemia, low birth weight, and preterm delivery, which made it more difficult to adjust the odds ratio of the number of parasite clones per infection on these pregnancy outcomes in regression analysis.

## Conclusions

These results indicate that the genetic diversity of *P. falciparum* is high in pregnant women from southern Brazzaville of the Republic of Congo, and the MOI might represent a risk for maternal anaemia.

## Data Availability

Datasets used and/or analysed during the current study are available from the corresponding author on reasonable request.

## Abbreviations

ACT: Artemisin-based Combination Therapy
CSA: Chondroitin sulfate A
DNA: Deoxyribonucleic acid
FCRM: Fondation Congolaise pour la Recherche Médicale
IPT: Intermittent preventive treatment
IQR: Interquartile range
ITNs: Insecticide treated bed nets
MOI: Multiplicity of infections
msp-1: Merozoite surface protein-1
PAM: Pregnancy associated malaria
PCR: Polymerase Chain Reaction
SD: Standard deviations
SP: Sulfadoxine-pyrimethamine
VSA: Variant surface antigens
